# Microbial Analyses of Subdural Empyema: A Case Report and Literature Review

**DOI:** 10.7759/cureus.76800

**Published:** 2025-01-02

**Authors:** Benjamin B Arbuckle, Rahim Abo Kasem, Adnan Shaik, Angela Downes, Steven Hwang, Peter G Passias, Nitin Agarwal, Andrew Jea, Muhammad Janjua

**Affiliations:** 1 Neurosurgery, University of Oklahoma Health Sciences Center, Oklahoma City, USA; 2 Neurological Surgery, Medical University of South Carolina, Charleston, USA; 3 Neurological Surgery, University of Missouri Kansas City School of Medicine, Kansas City, USA; 4 Neurosurgery, University of Colorado Anschutz Medical Campus, Aurora, USA; 5 Orthopedic Surgery, Shriners Children's Philadelphia, Philadelphia, USA; 6 Orthopedic Surgery, Duke University, Durham, USA; 7 Neurological Surgery, University of Pittsburgh Medical Center, Pittsburgh, USA; 8 Neurological Surgery, University of Oklahoma Health Sciences Center, Oklahoma City, USA

**Keywords:** antibiotics, case report, microbes, subdural empyema, surgical evacuation

## Abstract

Subdural empyema (SDE) is an infection between the dura and arachnoid mater, presenting with symptoms such as fever, headache, altered sensorium, neurological deficits, and seizures. Due to its rapidly progressive nature, early diagnosis and treatment are crucial. This paper aims to identify common pathogens, imaging findings, and the necessity of emergent neurosurgical intervention. We present the case of a 49-year-old woman with an SDE who underwent craniotomy to evacuate pus and hematoma. Cultures confirmed *Streptococcus intermedius,* and the patient improved postoperatively with the complete evacuation of the SDE and resolution of midline shift seen on the CT scan. A PubMed literature review focused on consolidating data to identify common pathogens, demographic details, treatment methods and duration, and outcomes in SDE. SDE requires prompt diagnosis and treatment. Contrast-enhanced brain MRI is crucial for diagnosis, showing features distinct from subdural hematoma or hygroma. Neurosurgical intervention is urgent, including craniotomy and evacuation. Postoperative broad-spectrum antibiotic therapy is essential until specific pathogens are identified, and a multidisciplinary approach is recommended.

## Introduction

Subdural empyema (SDE) is a purulent fluid collection between the dura mater and arachnoid meningeal layers encasing the brain. Purulent infection in this potential space is life-threatening and is associated with high rates of morbidity. SDE accounts for 20% of all intracranial infections and is more common among males, especially between the ages of 10 and 30 [1-3]. The etiology of SDE can vary and depends largely on an individual’s age and associated co-morbidities. Common organisms include anaerobic and microaerophilic streptococci. SDEs can arise from hematogenous spread, spread from meningitis, direct invasion from mastoid infections, trauma, and/or after transnasal or cranial surgery [1]. In infants, the infection most often occurs as a complication of meningitis [4,5]. The most common etiology in older children and adults is the spread of an infection of the paranasal sinuses, particularly the frontal sinus [2]. Seeding of the subdural space from a sinus infection can occur through direct extension through bone and dura mater, or the infection can gain access to the cranium through the valveless diploic veins, often accompanied by thrombophlebitis [1-3]. Rapid progression occurs once the infection has invaded the subdural space [2,6]. The resulting inflammation can cause irritation of the meninges, direct damage or compression of the brain tissue and the superficial cerebral veins, increased intracranial pressure, and mass effect [1,2].

The presentation for these cases is typically nonspecific. Rapid progression of the disease and rapid deterioration of the patient aid in diagnosis. Patients may present with headache, lethargy, and altered mental status. Physical exam may reveal fever, focal neurological deficit, and symptoms of meningitis such as neck stiffness and papilledema [1-3,6,7]. Seizure and status epilepticus are possible [1,6]. Prompt identification of the responsible pathogen from intraoperative cultures is crucial in guiding proper treatment to reduce morbidity and mortality. While many bacteria have traditionally been associated with an SDE, common pathogens implicated include anaerobic Gram-positive cocci and anaerobic Gram-negative bacilli such as *Staphylococcus aureus*, *Haemophilus influenzae*, and many *Streptococcus* species [1-3].

The treatment of SDE largely depends on the patient’s risk factors, diagnostic imaging, offending pathogen, and severity of symptoms. Long-term antibiotic therapy and emergent evacuation after craniotomy are key treatment modalities [1-3]. The primary objective of this paper is to present the common causes of SDE, how these cases can go undetected, the common pathogens involved, and the urgency of treatment. Neurosurgical intervention is detailed and discussed. A review of the literature to describe common pathogens is also included. We present a unique case of a 49-year-old woman with an SDE and a coexisting brain abscess. 

## Case presentation

**Figure 1:**
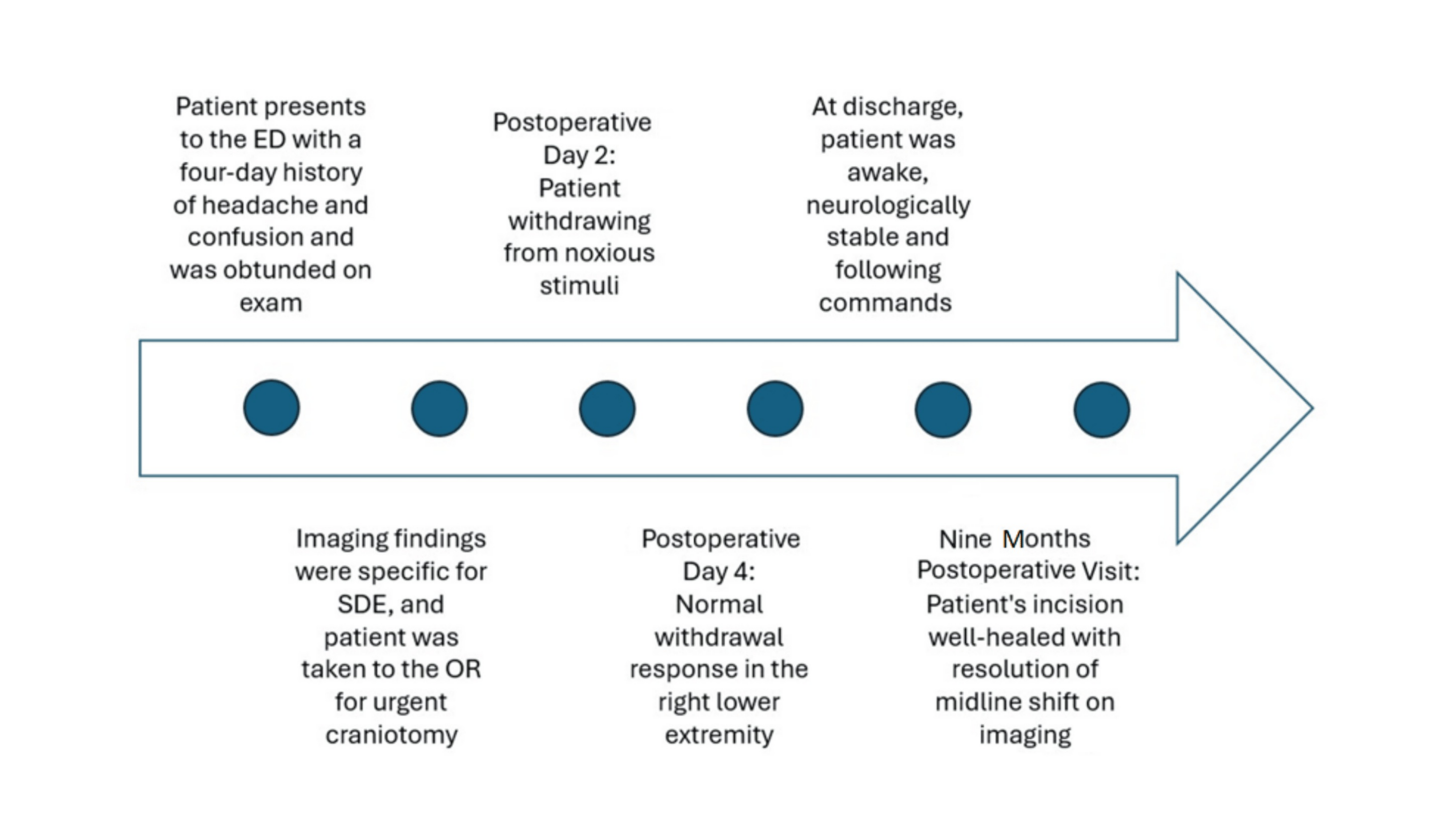
Clinical course timeline.

A 49-year-old woman with a past medical history of type II diabetes mellitus and right great toe osteomyelitis with recent amputation presented to the emergency department with complaints of a four-day history of headache and confusion. Upon examination, the patient had manifestations of obtundation, accompanied by left-sided hemiplegia and ptosis. Figure 1 presents a timeline detailing this episode of care. An initial CT scan and follow-up brain MRI without contrast showed a right-sided, hemispheric, thin, isointense collection. During a detailed neurological examination, the patient had bilateral eyes deviated to the right and downwards, exhibited facial grimace, and withdrew solely to painful stimuli in the left upper extremity. There was no response to painful stimuli in the right upper extremity or bilateral lower extremities.

**Figure 2:**
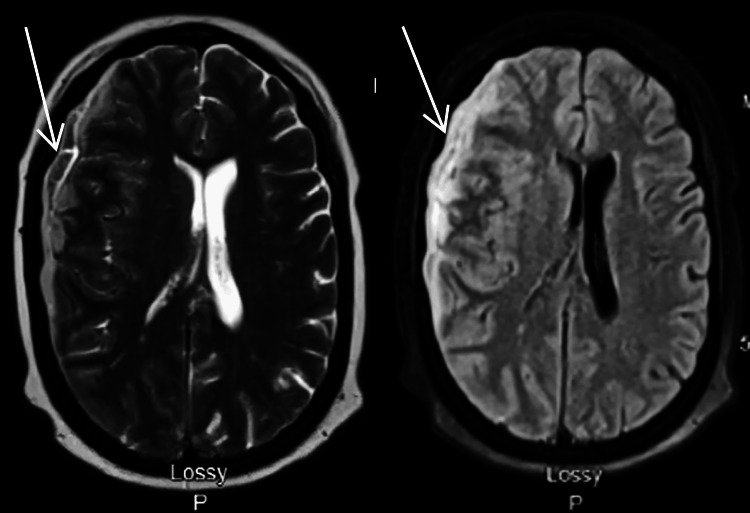
Contrast-enhanced brain MRI prior to surgery, demonstrating a thick-walled, peripherally enhancing, enlarging subdural collection overlying the right frontotemporal regions.

**Figure 3:**
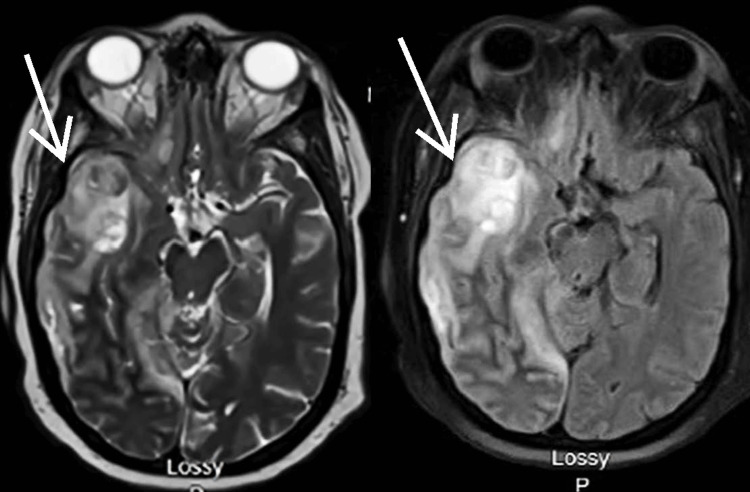
FLAIR sequence brain MRI, demonstrating the right temporal lobe hemorrhagic infarct with avid peripheral enhancement. FLAIR: fluid-attenuated inversion recovery.

A contrast-enhanced brain MRI showed a thick-walled, peripherally enhancing, enlarging subdural collection overlying the right frontotemporal regions (Figure 2). The collection demonstrated restricted diffusion, seen with acute blood products or infection. However, thick peripheral enhancement supported the diagnosis of an abscess or empyema. In addition, the right temporal lobe hemorrhagic infarct also showed an avid peripheral enhancement, further supporting the diagnosis of an abscess (Figure 3). Adjacent parenchymal edema, either inflammation or encephalitis, is best visualized on the fluid-attenuated inversion recovery (FLAIR) sequence (Figure 3). Given the imaging findings were highly specific for SDE, an urgent craniotomy was planned.

**Figure 4:**
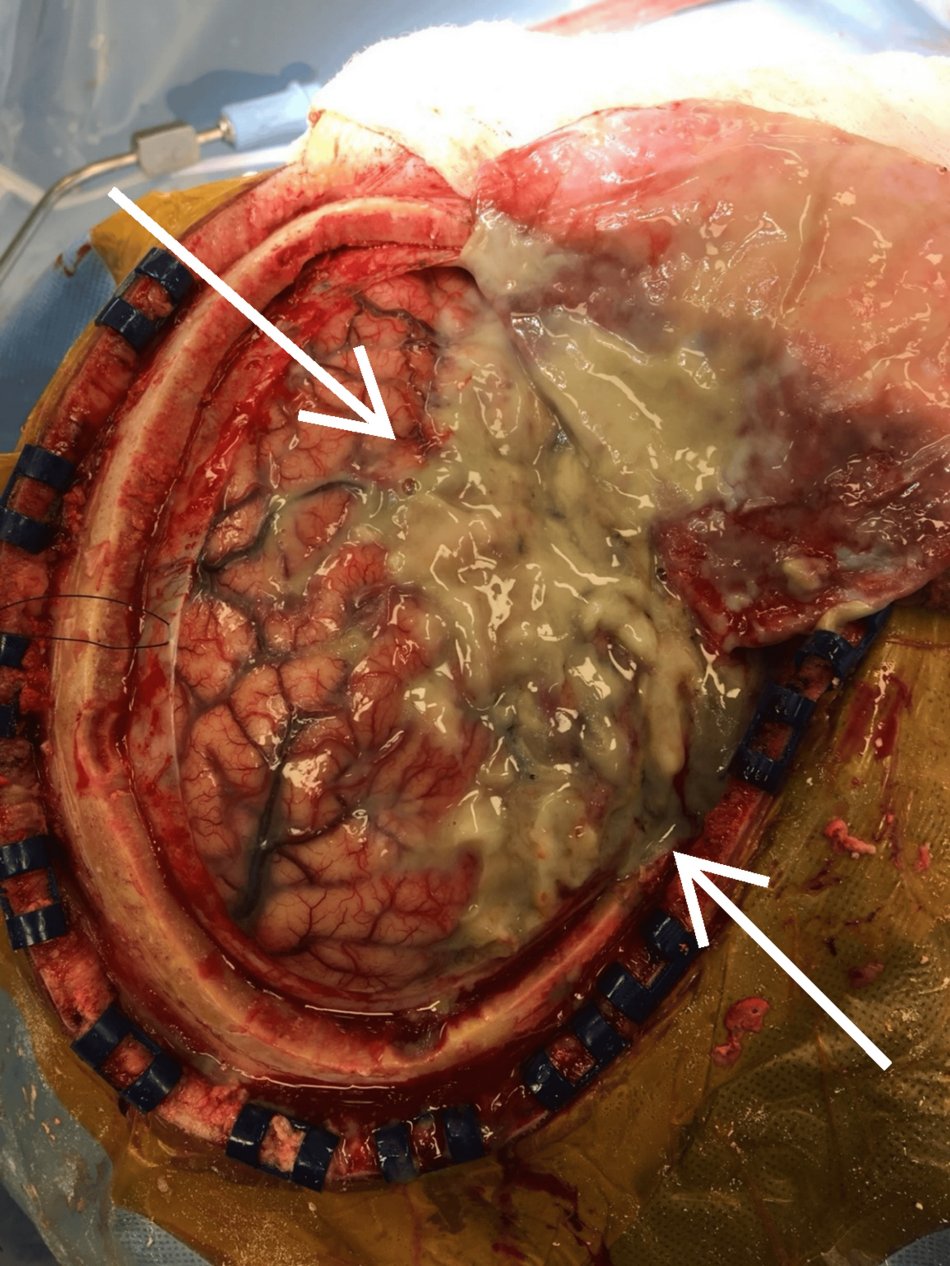
Intraoperative exposure of SDE showing pressured gross pus draining from the subdural space upon anterior reflection of the dura flap. SDE: subdural empyema.

After proper positioning and draping, a right-sided trauma flap incision was marked. The scalp flap was reflected anteriorly. Four burr holes were made, and a large bone flap was raised. The dura was opened first in the anterior frontal region using a Nurolon 4-0 holding stitch. Upon passing a Woodson under the dura, an egress of frank pus-stained cerebrospinal fluid (CSF) was appreciated. Four separate cultures (aerobic, anaerobic, fungal, and an acid-fast bacillus (AFB)) were taken and sent to the laboratory. The durotomy was further extended posteriorly parallel to the scalp and craniotomy flap. As soon as the dural flap was reflected anteriorly, frank pus poured out under pressure from the subdural space (Figure 4). 

Gentle irrigation was used, and the pus was slowly evacuated. A significant amount of phlegmon was encountered and appeared to be adherent to the cerebral cortex, especially in the frontotemporal region and deep in the Sylvian fissure all the way to the greater and the lesser wing of the sphenoid bone. Next, attention was given to the right frontal and temporal regions to evacuate the pus using constant gentle irrigation. Once the pus was thoroughly evacuated, the subtemporal region was entered. At this point, the necrotic inferior temporal gyrus was opened after a corticectomy, and deep-seated pus with a loculated hematoma was encountered, which was gently irrigated and suctioned out while avoiding any injury to the cortical veins. A sample of the necrotic, pus-stained tissue was sent for permanent biopsy. Two liters of normal saline were then used to irrigate all crevices thoroughly and to ensure a complete washout. A second look was given for any residual deep-seated phlegmon or loculation. Any obvious ooze was lined with Surgicel, a hemostatic agent. The meningeal dura was further inspected for any residual subdural membrane or phlegmon. The dura was approximated with the Nurolon 4-0 in an interrupted fashion, with ample gaps left for the delayed egress of any residual subdural fluid. The bone flap was placed back on and affixed with the use of titanium plates and screws. The scalp flap was reflected, and the incision was closed in layers. The patient was brought to the ICU and started on mechanical ventilation. 

Follow-up and outcomes 

The patient’s neurological exam improved postoperatively. Postoperative head CT scan without contrast revealed complete evacuation of the extensive right holo-hemispheric SDE and temporal lobe abscess with resolution of the midline shift. Cultures confirmed *Streptococcus intermedius* on postoperative day 5, and IV antibiotics were switched to ceftriaxone. Postoperative maxillofacial CT scan revealed maxillary premolar periapical abscesses; therefore, oral surgery was consulted, and tooth extraction was performed. 

On postoperative day 2, the patient began withdrawing from noxious stimuli in her left upper and lower extremities. By day 4, a normal withdrawal response was obtained in her right lower extremity. At discharge, the patient was neurologically stable and awake, and she followed oral commands with hand squeeze in the bilateral upper extremities and purposeful movement in her left upper extremity. Subsequently, the patient recovered dramatically well by nine-month follow-up. The scalp incision healed nicely. A head CT scan without contrast revealed no residual intra- or extra-axial collection with complete resolution of midline shift. 

## Discussion

Our case demonstrated one of the common etiologies of SDE. *Streptococcus anginosus* is a common cause of both infective endocarditis and SDE, requiring a high index of suspicion for an infectious etiology. Although *Streptococcus* species are among the most common etiologies of SDE, a literature review was necessary to describe the diversity and prevalence of infectious etiologies. 

**Figure 5:**
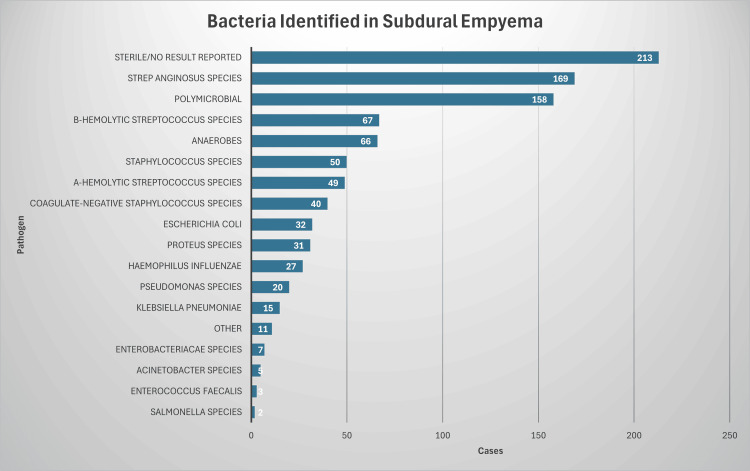
Distribution of bacterial pathogens identified in SDE cases. This bar graph elucidates the prevalence of bacterial species identified in clinical cases of SDE, with a conspicuous predominance of *Streptococcus anginosus* species (17.5%). Noteworthy is the proportion of cases where cultures remained sterile or yielded no results (22.1%), underscoring potential challenges in pathogen identification. SDE: subdural empyema.

The authors identified 12 articles composed of 1104 cases of SDE. Mortality data were universally reported across the 12 studies, and the overall mortality rate was 10.4%. Gender was specified in 1077 cases, with 65.2% of male patients. The average age was 21.0 years. In 965 cases, culture data were provided. Commonly identified pathogens were *S. anginosus* species (17.5%), β-hemolytic *Streptococcus* species (6.9%), and anaerobes such as *Bacteroides* and *Peptostreptococcus* species (6.8%). Some other pathogens identified included *Staphylococcus aureus* (5.2%), α-hemolytic *Streptococcus* species (5.1%), coagulase-negative *Staphylococcus* species (4.1%), *Escherichia coli* (3.3%), *Proteus* species (3.2%), *Haemophilus influenzae* (2.8%), *Pseudomonas* species (2.1%), and *Klebsiella pneumoniae* (1.6%). Sterile cultures (22.1%) and polymicrobial infections (16.4%) were also commonly reported (Figure 5) [8-19]. 

In the five studies specific to pediatric populations, 168 cases were identified. The mortality rate was 7.1%. Although data regarding sex and age were not provided in all studies, the mean age was 10.7 years, and 77.3% of patients were male. Bacteriology was reported for 167 cases, as one patient died before any intervention or cultures were drawn. *Streptococcus anginosus* species were responsible for 15.0% of infections. Other common pathogens identified in pediatric-specific studies included α-hemolytic *Streptococcus *species (9.6%), β-hemolytic *Streptococcus* species (6.0%), anaerobes (6.0%), *E. coli* (5.4%), *Proteus* species (4.8%), *Pseudomonas* species (4.8%), coagulase-negative *Staphylococcus* species (3.6%), *S. aureus* (3.0%), and polymicrobial infection (6.6%). Sterile cultures were reported in 29.3% of cases [10-12,17,18]. 

Rapid diagnosis and treatment of SDE are crucial for a patient’s survival [2,3,7]. The 10.4% mortality rate found in this literature review highlights this infection's treatment challenges. Prompt diagnosis is critical to guide targeted therapy. Identifying common SDE presentations in young adults, children, and males is crucial due to the higher prevalence in these groups [3]. Symptoms include headache, fever, neck stiffness, seizures, neurologic deficits, and altered mental status. Physical exam may show papilledema, cranial nerve palsies, contralateral hemiparesis, deteriorating level of consciousness, and signs of increased intracranial pressure [2,3]. Risk factors include orodental infection, sinusitis, recent trauma, and neurologic surgery [7]. Infants with meningitis, as well as children and young adults with sinusitis, head trauma, or a history of recent cranial surgery, are at risk for intracranial infectious complications such as SDE [2,4,5]. 

Upon clinical suspicion, imaging is essential to diagnose the infection. The empyema can often be viewed on a head CT scan, but an MRI brain scan with and without contrast is more sensitive [2]. Essential imaging findings are rim enhancement and restricted diffusion of the purulent subdural T2 bright fluid collection. Adjacent encephalitis will likely be seen. The MRI brain scan can also assess the degree or severity of midline shift and sequelae of infection, such as adjacent abscess, stroke, and venous sinus thrombophlebitis. 

Building on the existing literature, Jolayemi et al. echo our literature review findings, showing a predominance of SDE in younger males (mean age of 21.37 years, M:F ratio of 2.5:1), consistent with our data [20]. Additionally, Jolayemi et al. reported a 16.3% mortality rate, comparable to our 10.4%, but their cultures were 95.9% sterile, posing challenges for antibiotic optimization. The majority of patients in this cohort underwent surgical treatment via burr hole drainage and washout. Jolayemi et al. highlight the potential limitations of this surgical approach, noting an increased risk of postoperative residual SDE in their burr hole group compared to the craniotomy group. Analyzing the specimens is a critical step in guiding management options for SDE. The literature reports a wide range of positive culture rates, from 54% to 81%, consistent with the 77.9% observed in our series [1]. This reflects similar studies in our review, although others have reported lower culture positivity [4,8,9,15,19]. The spectrum of pathogens identified in SDE commonly encompasses species such as *Streptococcus *spp., *Staphylococcus* spp., *H. influenzae*, in addition to less frequent organisms such as *Proteus mirabilis*, *Salmonella typhi*, and *E. coli* [5,21,22]. In concordance with these findings, *Streptococcus* spp. accounted for 29.5% of pathogens in our analysis, with significant occurrences of *Staphylococcus* spp., *H. influenzae*, *Proteus mirabilis*, and *E. coli*. Notably, our data showed a minimal presence of *Salmonella* spp. at 0.2%, deviating from the general trend. 

Broad-spectrum antibiotics are a mainstay of treatment because of the many bacteria implicated in the literature. As previously mentioned, bacteria that must be covered by antibiotic selection include *S. anginosus* species, α-hemolytic and β-hemolytic *Streptococcus* species, coagulase-positive and coagulase-negative *Staphylococcus* species, anaerobes, and *E. coli*. If available, susceptibility from bacterial cultures should further guide antibiotic selection; however, cultures from an SDE yield no results between 7% and 52% of the time, possibly because antibiotics have often already been given [23]. Antibiotics should be given intravenously for at least two weeks, followed by four to six weeks of oral administration [1-3,5]. A third-generation cephalosporin with metronidazole is a common combination for the antibiotic regimen used [2,23]. Antiepileptic therapy is also reasonable to initiate due to the associated high risk of seizures [6]. Each of these therapies helps reduce morbidity and mortality and should be coupled with urgent surgery to evacuate the pus and to provide the best possible outcomes. 

Wide craniotomy with complete evacuation is the preferred surgery for SDE. While burr holes are sometimes considered an alternative, craniotomy has been shown to have lower rates of reoperation and is therefore preferred [9]. Burr holes have also been demonstrated to have an increased risk of outcomes relative to craniotomy [20,24]. Long-term morbidity is still common, and roughly half of patients will suffer residual neurological deficits. Herniation and level of consciousness at presentation are factors that impact the likelihood of long-term complications, as is the timeliness of treatment, highlighting the importance of rapid diagnosis and intervention [2,3]. 

Given that a number of these patients present with focal neurological deficits, rehabilitation is a key component of care and should start as early as possible [20]. Inpatient or outpatient services may be needed for speech and physical rehabilitation [3]. Speech and swallow evaluations are routinely performed to assess swallowing, and consultation can be requested for gastric tube placement. Rehabilitation often begins with inpatient rehabilitation using Hoyer lifts, progressing to the need for assisted standing devices, followed by hand-driven or motorized wheelchairs, and, finally, walkers. While rehabilitation can be acute, prolonged long-term rehabilitation may be indicated if the diagnosis of SDE is delayed and the patient develops substantial functional loss, such as hemiparesis [25,26]. 

There are some limitations of this study. The study design is retrospective, so the results are based on previously published studies. In addition, as a case report, we are limited by a lack of data analysis. Most of the studies did not mention a detailed microbial analysis and lacked a true association between microbes and the choice of antibiotics instituted. Selection bias from previously published case studies cannot be eradicated. Moreover, the described techniques and pearls are learned through experience from the experts in the field. Future analysis through patient database reviews and/or operative video databases can lead to more robust results and potential augmentation of SDE guidelines. 

## Conclusions

A thorough neurological examination, systemic workup, and MRI of the brain with and without contrast are needed for an early diagnosis of SDE. Early source control, culture, and sensitivity-driven broad-spectrum antibiotics are prudent. The duration of antibiotics is determined after consultation with the infectious disease; therefore, a multispecialty approach is instituted. With early recognition, antibiotic therapy, and surgical evacuation, the patient in this study had a promising recovery with minimal long-term neurologic sequelae. Referral to an acute rehabilitation unit is warranted to start early and prolonged physical and occupational therapies to enhance optimal neuromuscular recovery. The outcome of the presented case is evidence of prompt recognition and treatment of SDE.
